# Horner syndrome as a manifestation of an exacerbation of Takayasu’s arteritis

**DOI:** 10.1186/1546-0096-10-S1-A84

**Published:** 2012-07-13

**Authors:** Dominic Co, James Nocton

**Affiliations:** 1 Medical College of Wisconsin, Milwaukee, WI, USA

## Purpose

Takayasu’s arteritis is a chronic, granulomatous, large vessel vasculitis affecting the aorta and its large branches, and occurring most commonly in young women. It is the third most frequently diagnosed vasculitis in childhood. Generally, symptoms reflect ischemia to an affected organ or limb. A small percentage of patients develop neurologic symptoms such as visual disturbances, transient ischemic attacks (TIA’s) or stroke. Horner syndrome is an unusual manifestation of Takayasu’s, with only a single reported case of an adult patient in whom Horner syndrome was the presenting manifestation of Takayasu’s arteritis. We present a 14 year old with a history of Takayasu’s arteritis who developed Horner syndrome during the course of the illness.

## Results

### Case report

A 14 year old female was diagnosed at the age of 11 years with Takayasu’s arteritis after developing signs and symptoms of acute congestive heart failure while receiving fluid resuscitation for dehydration. She had a history of mild fatigue, decreased endurance, and symptoms of claudication. She was found to have complete occlusion of the superior mesenteric artery and involvement of the celiac, splenic, hepatic, right subclavian, and right renal arteries along with cardiomyopathy. She was treated with prednisone, methotrexate and infliximab with improvement in her symptoms, and stabilization of her cardiomyopathy without signs of progressive vasculitis on serial magnetic resonance imaging studies (MRI/MRA). Shewas in stable condition two years after her diagnosis, treated with infliximab every 6 weeks and methotrexate, when she developed left shoulder pain and left conjunctival injection. On ophthalmologic exam, she was noted to have episcleritis of the left eye as well as ptosis of her left upper eyelid and miosis of her left pupil without anhidrosis (Horner syndrome). An MRI/MRA of her brain and neck revealed stable narrowing of her right subclavian but no masses, no new aneurysms or stenoses, and no evidence of new overt inflammation. Her ESR was 16 mm/h and CRP 0.6 mg/dL (prior baseline 15-17 mm/h and 0.3-0.6 mg/dL, respectively). Given the lack of anhidrosis, her lesion was localized to the superior cervical ganglion. In the absence of other causes, and with the concurrent development of episcleritis, the Horner syndrome was presumed secondary to an exacerbation of vasculitis. Prednisone was started at 40mg orally once daily, followed by a slow taper, and infliximab and methotrexate were continued. Within 1 week the episcleritis resolved, and within 3 weeks, the ptosis and miosis had nearly completely resolved.

## Conclusion

Takayasu’s arteritis can result in a wide variety of non-specific signs and symptoms, including a variety of neurologic manifestations. To our knowledge, this is the first reported case of Horner syndrome in a child due to Takayasu’s arteritis. Takayasu’s arteritis should be considered in the differential diagnosis of Horner syndrome.

## Disclosure

Dominic Co: None; James Nocton: None.

